# Absorption of Iodide of Potassium by the Skin

**Published:** 1870-03

**Authors:** 


### Absorption of Iodide of Potassium by the Skin.

Dr. Ferrand refers to a case of iodism occuring in private practice, as a consequence of the application to the skin of dry iodide of potassium. This fact, besides showing the possibility of the absorption of dry substances by the skin, affords a new means of administration of this important remedy. The case was that of a female who was attacked by inflammatorv symptoms in the pelvic region, accompanied with purulent evacuations through the rectum. After a careful diagnosis, it was established that these symptoms were due to the presence of an immense pelvic abscess, occupying all the space behind the uterus and the broad ligaments. Dr. Ferrand wished to combine with the local symptomatic treatment the internal use of iodide of potassium in pretty strong doses; but in a few days very distinct symptoms of iodism obliged him to desist, although the fitness of the remedy appeared already manifested by a sensible improvement in the condition of the patient. Scarcely had the iodic phenomena ceased, when the readministration of the iodide appeared to be justified; but it became necessary to suspend it again after three days. It was then that Dr. Ferrand thought of the application of dry iodine of potassium. With this view he made the patient put on a shirt which had been previously dipped in a solution of two drachms of iodide, and then dried. After three days this shirt was replaced by a second, prepared in the same manner; but towards the end of the fourth day the iodic symptoms recurred. These symptoms were coryza, lachrymation, a sense of weight and pain over the frontal sinews, a sense of occlusion of the posterior nares, muscular pain extending over the whole region of the neck, constant nausea accompanied with frequent vomiting, and smart fever. All these phenomena corresponded, in fact, with those previously observed during the internal iodic treatment; and although they appeared after a longer lapse of time, when the remedy was applied externally, yet they remained more pertinaciously, showing that if the action of the medicine were a little less prompt, it acted more deeply and persistently than was the case when administered according to the usual method.—*Edinburgh Medical Journal*, Nov., 1869, from *Bull. Gen de Therap.*—*American Medical Journal.*

